# AGD1/USP10/METTL13 complexes enhance cancer stem cells proliferation and diminish the therapeutic effect of docetaxel *via* CD44 m6A modification in castration resistant prostate cancer

**DOI:** 10.1186/s13046-025-03272-3

**Published:** 2025-01-14

**Authors:** Hong Wang, Chunli Cui, Weiyi Li, Hui Wu, Jianjun Sha, Jiahua Pan, Wei Xue

**Affiliations:** 1 Department of Urology, Ren Ji Hospital, Shanghai Jiao Tong University School of Medicine, Shanghai, China; 2 Department of Special Examinations, Qingdao Women and Children Hospital, Qingdao, China; 3 Department of Urology, Shanghai East Hospital, School of Medicine, Tongji University, Shanghai, China

**Keywords:** AGD1, USP10, METTL13, m6A, Ubiquitination

## Abstract

**Background:**

Most patients with prostate cancer inevitably progress to castration-resistant prostate cancer (CRPC), at which stage chemotherapeutics like docetaxel become the first-line treatment. However, chemotherapy resistance typically develops after an initial period of therapeutic efficacy. Increasing evidence indicates that cancer stem cells confer chemotherapy resistance via exosomes. This study demonstrated that AGD1, derived from prostate cancer stem cells (PCSCs), enhanced the stemness of prostate cancer cells and reduced the therapeutic effect of docetaxel in CRPC.

**Methods:**

Quantitative real-time PCR (qPCR) was employed to determine the expression levels of AGD1 and METTL13 mRNAs in PCSCs and exosomes. Protein expression levels were examined using western blots and dot blots. The potential functions of AGD1 and METTL13 in CRPC were investigated through cell proliferation assay, Transwell assay, EdU incorporation assays, Annexin V-FITC/PI staining, and sphere formation assays. To uncover the underlying mechanisms of AGD1, RNA pull-down assay, RIP, co-Immunoprecipitation (co-IP), mass spectrometry (MS), Methylated RNA immunoprecipitation (MeRIP) and single-base elongation and ligation-based qPCR amplification method (SELECT) were performed. The effects of AGD1 and METTL13 on CRPC development and metastasis under docetaxel treatment were analyzed using a xenograft mouse model and an organoid model. Additionally, liposomal-chitosan nanocomplex drug delivery systems were designed to explore AGD1’s role in regulating docetaxel treatment resistance in CRPC.

**Results:**

AGD1 expression was upregulated in PCSCs and exosomes. Downregulating AGD1 enhanced the sensitivity of CRPC to docetaxel treatment by inhibiting their stemness, with the reverse also being true. RNA pull-down, combined with MS, co-IP and RIP assays, demonstrated that AGD1 binds to METTL13 and USP10, forming a complex that facilitates METTL13 protein accumulation through USP10-induced deubiquitination. MeRIP assay and SELECT assay revealed that METTL13 transcriptionally controls the mRNA decay of CD44 via m6A methylation. Additionally, this process activates the pSTAT3/PI3K-AKT signaling pathway. Organoid models and liposomal-chitosan nanocomplex drug delivery systems showed that reducing AGD1 expression enhanced the therapeutic effect of docetaxel in CRPC.

**Conclusions:**

AGD1 mediates the stemness and apoptosis of PCSCs and promotes docetaxel treatment resistance by enhancing tumor growth and metastasis through USP10/METTL13-mediated CD44 mRNA decay in CRPC.

**Supplementary Information:**

The online version contains supplementary material available at 10.1186/s13046-025-03272-3.

## Introduction

According to the latest global statistics, prostate cancer (PCa) has become the leading cause of morbidity and mortality among older men in the urinary system [1]. Rapid progression to castration-resistant prostate cancer (CRPC), a critical stage in PCa, significantly shortens overall survival (OS) [2, 3]. Following the latest AUA & EUA guidelines, chemotherapeutics such as docetaxel are the first-line treatment options at this stage [2, 4]. However, most patients inevitably develop treatment resistance, and restoring the chemotherapy sensitivity of docetaxel would benefit patients with PCa. Cancer stem cells (CSCs), a small population of heterogeneous cells within tumors, possess pluripotent properties. Previous reports suggest that CSCs contribute to chemotherapy and radiation resistance through mechanisms such as ROS defense, upregulation of drug efflux pumps, and enhanced DNA damage repair (DDR) systems [5–7]. Similar to CSCs, prostate cancer stem cells (PCSCs) are distinguished by markers such as CD44, CD133, CD49f, Integrins α2/β1, ALDH1, KLF4, SOX2, NANOG, and P63. Using these markers, PCSCs can be isolated from cell lines and surgical tissue *via* FACS or MACS [8]. Additionally, organoids, a 3D culture system supporting long-term expansion of PCa, facilitate the study of interactions between stem cells and cancer cells [9] and making them a valuable pre-clinical tool for screening novel therapeutic agents. In our study, we explored the role of AGD1 in the resistance treatment of docetaxel in CRPC via affecting the stemness of prostate cancer cells.

Normal stem cell niches suppress tumor growth, while CSCs niches promote malignant progression through various secretory microvesicles or exosomes [10, 11]. Increasing evidence suggests that CSCs confer chemotherapy resistance *via* exosomes [12–14]. Exosomes, ranging from 40 to 100 nm in size, play an integral role in carcinogenesis, malignant progression, and treatment resistance. They contain numerous mRNAs, non-coding RNAs, proteins, and signaling molecules that influence cancer cell fate [15, 16]. miRNA-host gene lncRNAs (lncmiRHGs), which contain miRNAs within exon or intron sequences, serve as promising diagnostic and prognostic biomarkers and play critical roles in cancer development [17]. AGD1, also named as MIR100HG, a subclass of lncmiRHG, encodes three miRNAs within introns: miR-125b-1, miR-100, and let-7a-2. Evidence shows that AGD1 is dysregulated in multiple tumors compared to normal tissues, acting as either an oncogene or tumor suppressor [18]. Previous studies reported that AGD1, together with miR-100 and miR-125b, activates Wnt signaling, inducing treatment resistance to cetuximab in colorectal cancer [19]. However, the function and biogenesis of lncmiRHGs remain largely unexplored. Understanding the mechanism of AGD1 could lead to new treatment strategies for PCa. In our study, we demonstrated that AGD1 derived from PCSCs exosomes promoted the treatment resistance of docetaxel in CRPC.

Post-translational modifications (PTMs) regulate gene expression at the post-transcriptional level, affecting protein activity, degradation and function. Ubiquitination and de-ubiquitination are widespread and reversible PTMs that interact with various proteins to regulate many biological processes [20]. Enzymes involved in ubiquitination and de-ubiquitination either attach or remove ubiquitin, respectively. USP10, a de-ubiquitinating enzyme, removes ubiquitin from substrate proteins, thereby regulating numerous biological processes [21, 22]. Previous researches have shown that USP10 plays a crucial role in breast cancer progression, lung tumorigenesis, glioblastoma cell apoptosis, gastric cancer development, and chemoresistance in Notch1-hyperactivated pancreatic cancer [22–25]. In our study, USP10 was found to remove ubiquitin from METTL13, promoting the accumulation of METTL13 protein and diminishing the therapeutic effect of docetaxel in CRPC. N6-methyladenosine (m6A) modification is another PTM at the RNA level that participates in various biological and pathological processes [26]. METTL13, the “writer” enzyme of m6A, catalyzes m6A modifications on specific RNA. Previous studies reported that METTL13 promoted various cancers’ development but inhibited the progression of clear cell renal cell carcinoma and bladder cancer [27, 28]. The role and mechanism of METTL13 in PCa remain unexplored, making the investigation of METTL13 crucial for understanding PCa progression.

This study aims to clarify the function of AGD1 derived from PCSCs both in vitro and in vivo, including using a PCa organoid model and nano-drug delivery systems (NDDS). Our research demonstrated that AGD1 promotes docetaxel treatment resistance in CRPC through paracrine and distant secretion. Furthermore, the interaction between AGD1, USP10, and METTL13 was elucidated, which forms a complex that upregulates the stemness of PCa cells, contributing to docetaxel resistance. In summary, these results highlight a potential therapeutic target for CRPC.

## Materials and methods

### Patient samples and cell culture

All patients provided informed consent in accordance with the Ethics Committee of Shanghai Jiao Tong University School of Medicine and the Renji Hospital Ethics Committee (KY2022 136 A). Detailed patient information is listed in Supplementary Table S1. All cell lines were obtained from the Shanghai Chinese Academy of Sciences (Shanghai, China). PCa cell lines, including PC3, DU145, and 22RV1, were cultivated in RPMI-1640 medium (Gibco, USA) supplemented with 10% fetal bovine serum (FBS, Gibco) and 1% penicillin/streptomycin (Yesean, Shanghai, China). Human normal prostate epithelial cells (RWPE-1) were cultured in a Defined Keratinocyte SFM medium (Gibco). PCSCs were cultured in serum-free DMEM/F12 medium (Gibco) supplemented with 20 ng/ml epidermal growth factor (EGF, Sigma, Saint Louis, USA), 20 ng/ml basic fibroblast growth factor (bFGF, Sigma), 0.4% bovine serum albumin (BSA, Sigma), 5 µg/ml insulin (Sigma), and N2 supplement (Stemcell Technologies Inc., Canada) as previously described [29].

### Western blots

PCa cells were lysed in RIPA lysis buffer for 30 min on ice, and protein concentrations were measured using the BCA Protein Assay (Beyotime Biotechnology, Shanghai, China). Each sample contained 30 µg of protein, loaded onto SDS-PAGE gels (Bio-Rad Laboratories) and transferred to PVDF membranes (Sigma-Aldrich). Following a 1-hour block with 5% BSA at room temperature, the membranes were incubated with primary antibodies overnight at 4 °C. The primary antibodies, listed in Supplementary Table S2, included CD133, CD44, SOX2, KLF4, TSG101, ALIX, CD9, Calnexin, METTL13, USP10, AKT, p-AKT, ERK1/2, p-ERK1/2, NFκB, p-NFκB, and GAPDH. After extensive washing with PBST, the membranes were incubated with HRP-conjugated secondary antibodies for 1 h at room temperature. Subsequent washes with PBST preceded chemiluminescence detection (Cell Signaling Technology) using the Odyssey two-color infrared laser imaging system (LI-COR Biosciences, Lincoln, NE, USA).

### RNA isolation and real-time PCR

Total RNA was extracted from PCa cells using Trizol reagent (Yesean, China). Reverse transcription of cDNA was performed with the HiScript^®^ III 1st Strand cDNA Synthesis Kit (Vazyme, Nanjing, China), and real-time PCR was conducted using the ChamQ SYBR qPCR Master Mix Kit (Vazyme, China) according to the manufacturer’s instructions. Primers for the genes were synthesized by Sangon (Shanghai, China), with detailed information listed in Supplementary Table S2.

### Isolation and identification exosomes derived from PCSCs (PCSCs-exos)

The conditioned medium of PCSCs, cultured for 48 h, was collected and centrifuged at 3000 ×g for 15 min to remove dead cells and debris. After further centrifugation at 10,000 ×g for 30 min to remove extracellular vesicles (EVs), the supernatant was filtered through 0.22 μm filter membranes and ultracentrifuged at 100,000 ×g at 4 °C for 1 h. The pellet was then resuspended in PBS for exosome research [30]. Transmission electron microscopy (TEM) analyzed the size distribution of exosomes. The BCA Protein Assay Kit (Vazyme, China) quantified the protein concentration of exosomes. Exosomal markers TSG101, CD9, and ALIX, along with the negative marker calnexin, were detected *via* western blot. Additionally, CM-Dil dye (Yesean, China) labeled exosomes as described previously [31]. Briefly, PCa cells were incubated with CM-Dil-labeled exosome solution at 4 °C in the dark for 15 min. After washing three times with PBS, imaging analysis was performed using confocal laser scanning microscopy (TCS SP5, Leica).

### Lentivirus infection and cell transfection

TcDNA3.1-based vectors encoding the full-length cDNA of human AGD1 and METTL13, as well as control plasmids and short hairpin sequences against AGD1, METTL13, and a negative control, were constructed by Genomeditech (Shanghai, China). Detailed information about these plasmids is listed in Supplementary Table S2. Briefly, these plasmids were transfected into HEK-293T cells, and the resulting virus was used to infect PCa cells. The treated cells were selected with puromycin to obtain stable cell lines with gene knockdown or overexpression.

For cell transfection, Lipofectamine 3000 (Invitrogen, USA) was used to transfect the overexpression or shRNA plasmids into PCa cells according to the manufacturer’s instructions. Real-time PCR and western blot analyses were performed to evaluate the efficiency of overexpression or knockdown.

### Cell proliferation assay, EdU incorporation assay, colony formation assay, and flow cytometry assay

Cell proliferation was assessed using the cell proliferation assay kit (Yesean, Shanghai, China) and 5-Ethynyl-2′-Deoxyuridine (EdU) assay (Beyotime Biotechnology, China) following treatment with 20 nM docetaxel (Meilune, Dalian, China), according to the manufacturer’s instructions.

For the colony formation assay, 1,000 cells were seeded per well in 6-well plates and cultured for 14 days. The cells were then fixed with 75% ethanol and stained with 0.1% crystal violet, with images captured using a digital camera.

The Annexin V-FITC/PI assay was conducted using the Annexin V-FITC/PI KIT (Becton, Dickinson and Company, #556547, USA). Stemness of PCa cells was analyzed using the ALDEFLUOR™ assay kit (StemCell Technologies, Herndon, VA, USA). Treated cells were harvested, thoroughly washed with PBS, and incubated with the appropriate dye per the manufacturer’s instructions. After another PBS wash, the cells were analyzed on an LSRFortessa SORP (BD Biosciences, Franklin Lakes, NJ), and data were processed with FlowJo software (Ashland, OR). Three independent experiments were performed.

### Animal assays

Four-week-old male BALB/C nude mice, purchased from SLAC Laboratory Animal (Shanghai, China), were maintained under SPF conditions. Mice were randomly assigned to control and treatment groups. In the subcutaneous tumor experiment, 5 × 10^6^ PCa cells in 100 µL were injected subcutaneously into the right inguinal region. After one week, docetaxel (10 mg/kg) was administered *via* intraperitoneal injection twice weekly, with tumor sizes measured twice weekly using the formula: volume = 0.5 × length × width^2^. After one month, mice were sacrificed, and tumors were measured and fixed in 4% paraformaldehyde. For the pulmonary metastatic assay, 1 × 10^6^ luciferase-expressing PCa cells in 100 µL were injected into the tail vein of six-week-old nude mice. Docetaxel (10 mg/kg) was administered intraperitoneally twice weekly starting one week post-injection. After one month, mice were monitored weekly using an in vivo imaging system (IVIS), and metastatic foci were analyzed using aniView software. All animal experiments adhered to the Guide for the Care and Use of Laboratory Animals (National Academies Press, 2011) and were approved by the Animal Care Committee of Shanghai Jiao Tong University School of Medicine.

### Prostasphere formation

A total of 1,000 treated PCa cells per well were resuspended in 500 µL of DMEM/F12 medium supplemented with growth factors and cultured in 24-well ultra-low-attachment plates. The medium was refreshed every 2–3 days. After two weeks, the prostaspheres were measured and photographed using light microscopy. Prostaspheres with diameters greater than 100 μm were considered stem colonies. Three independent experiments were conducted.

### Organoid culture and lentiviral transduction

Organoids derived from patients with CRPC exhibiting docetaxel resistance were established based on previous reports [32, 33]. Lentiviral transduction of shRNA AGD1 and shRNA NC followed the previously described protocol [34]. Briefly, organoids were trypsinized, suspended in lentivirus-containing organoid media with 8 µg/mL polybrene, and incubated for 1 h at 37 °C before embedding in Matrigel. After 48–72 h of culture, organoids were selected with 1 µg/mL puromycin. The proliferation of organoids treated with docetaxel was analyzed on the fifth day using the cell proliferation assay. Three independent experiments were conducted.

### RNA pull-down assay, RNA immunoprecipitation (RIP) assay, and co-immunoprecipitation (co-IP) assay

An AGD1 pull-down assay was performed using an RNA pull-down kit (Bersinbio, Guangzhou, China) following the manufacturer’s instructions. AGD1 and control probes were designed and synthesized by GenePharma (Shanghai, China), with detailed information provided in Supplementary Table S2. Briefly, 10^7^ PCa cells were lysed on ice for 30 min and then sonicated. The probes were incubated with Streptavidin magnetic beads for 30 min at room temperature to obtain probe-coated beads, followed by incubation with protein extract under gentle rotation for 2 h at room temperature. After washing with RIP buffer, RNA-binding proteins (RBPs) were extracted for PAGE gel electrophoresis and immunoblotting. Silver-stained bands from the PAGE gel were cut out for further mass spectrometry (MS) analysis.

For the RNA immunoprecipitation (RIP) assay, the EZ-Magna RIP kit (Millipore, Billerica, MA, USA) was used to analyze the interaction between METTL13 and AGD1, according to the manufacturer’s recommendations. Briefly, cells were lysed in RIP lysis buffer and incubated with METTL13 antibody or IgG overnight at 4 °C. After washing with PBS, the RNAs co-precipitated by METTL13 were extracted and analyzed using real-time PCR.

To explore the binding proteins with METTL13, a Co-IP assay was conducted. Cells were lysed with buffer (Epizyme, Shanghai, China) on ice for 30 min and centrifuged at 12,000 rpm for 15 min to remove cellular debris. The protein extract was incubated with Dynabeads Protein G (Yesean, China) with gentle shaking at 4 °C for 1 h. Subsequently, the protein-bead complexes were incubated with 5 µg of METTL13 antibody on a shaker at 4 °C overnight. After washing three times with lysis buffer, the binding proteins and 10% inputs were detected by western blot.

### Immunocytochemistry and immunohistochemistry

Immunocytochemistry and immunohistochemistry involved fixing PCa cells or tissue sections with 4% paraformaldehyde (PFA) and permeabilizing with 0.5% Triton-X 100 (Sigma). Blocking was performed using 5% BSA for 1 h at room temperature, followed by overnight incubation with primary antibodies at 4 °C. After two PBS washes the next day, the samples were incubated with fluorescein isothiocyanate (FITC) or rhodamine-conjugated secondary antibodies (Sigma) for 1 h at room temperature. The nuclei were labeled with DAPI, and images were captured and analyzed using a fluorescence microscope (Nikon, Tokyo, Japan) or light microscope.

### m6A dot blot assay

The dot blot assay was conducted to analyze the total m6A RNA levels in treated cells. Total RNA was extracted and denatured at 95 °C for 3 min to disrupt secondary structures. RNA samples (400 ng, 200 ng, and 100 ng) were spotted on Amersham Hybond-N + membranes (GE Healthcare, USA) and crosslinked by UV for 30 min. After blocking with 5% BSA for 30 min at room temperature, the membranes were incubated with m6A antibody overnight at 4 °C. Following PBST washes, the membranes were incubated with HRP-conjugated secondary antibody. Images were captured and analyzed by chemiluminescence (Cell Signaling Technology) using an imaging system.

### Methylated RNA immunoprecipitation (MeRIP) and single-base elongation and ligation-based qPCR amplification method (SELECT)

Methylated RNA immunoprecipitation (MeRIP) was conducted using the MeRIP Kit (BersinBio, Guangzhou, China) following the manufacturer’s instructions [35]. Briefly, 300 µg of RNA per sample, extracted from the shAGD1 and control groups, was fragmented into approximately 300 bp segments. The RNA fragments were immunoprecipitated with m6A antibody (Abcam, ab208577) or immunoglobulin G (IgG) conjugated with protein A/G magnetic beads for 2 h at 4 °C. The 10% input mRNA and m6A-enriched mRNA were used to construct RNA sequencing libraries on a Novaseq sequencer (Illumina, USA) using the PE150 strategy. MeTDiff software was employed to analyze the MeRIP-seq data [36]. Specific position of m6A modification on CD44 3’UTR was probed by single-base elongation and ligation-based qPCR amplification (SELECT) as previously reported [37], and the detected sites and qPCR primers were listed in Supplementary Table S2.

### RNA stability assays

Following 24 h of plasmid transfection in PCa cells, the cells were co-cultured with 10 µg/mL actinomycin D (Yesean, China). Samples were collected at 0, 2, 4, 6, 8, and 10 h post-treatment. Total RNA was extracted, reverse transcribed, and analyzed by real-time PCR. Three independent experiments were conducted.

### Uptake of liposomal - chitosan/siAGD1 nanocomplexes

Nanocomplex uptake was examined using TEM. PC3 and DU145 cells were seeded in six-well plates and incubated overnight until reaching 60–70% confluence. Fresh medium containing 2 µM nanocomplexes was then added, and the cells were cultured for 6 h before being switched to fresh complete medium. The cells were subsequently harvested for in vivo studies and TEM analysis. For TEM, the cells were fixed overnight at 4 °C in 2.5% glutaraldehyde.

### Statistical analysis

Data were analyzed using GraphPad Prism 7 (San Diego, CA) and are presented as mean ± SD. Depending on the experiment type, Student’s t-test, one-way ANOVA, and Pearson correlation coefficients were employed. A p-value of < 0.05 was considered statistically significant. All experiments were performed in triplicate.

## Results

### AGD1 was upregulated in PCSCs and their exosomes

**Fig. 1:**
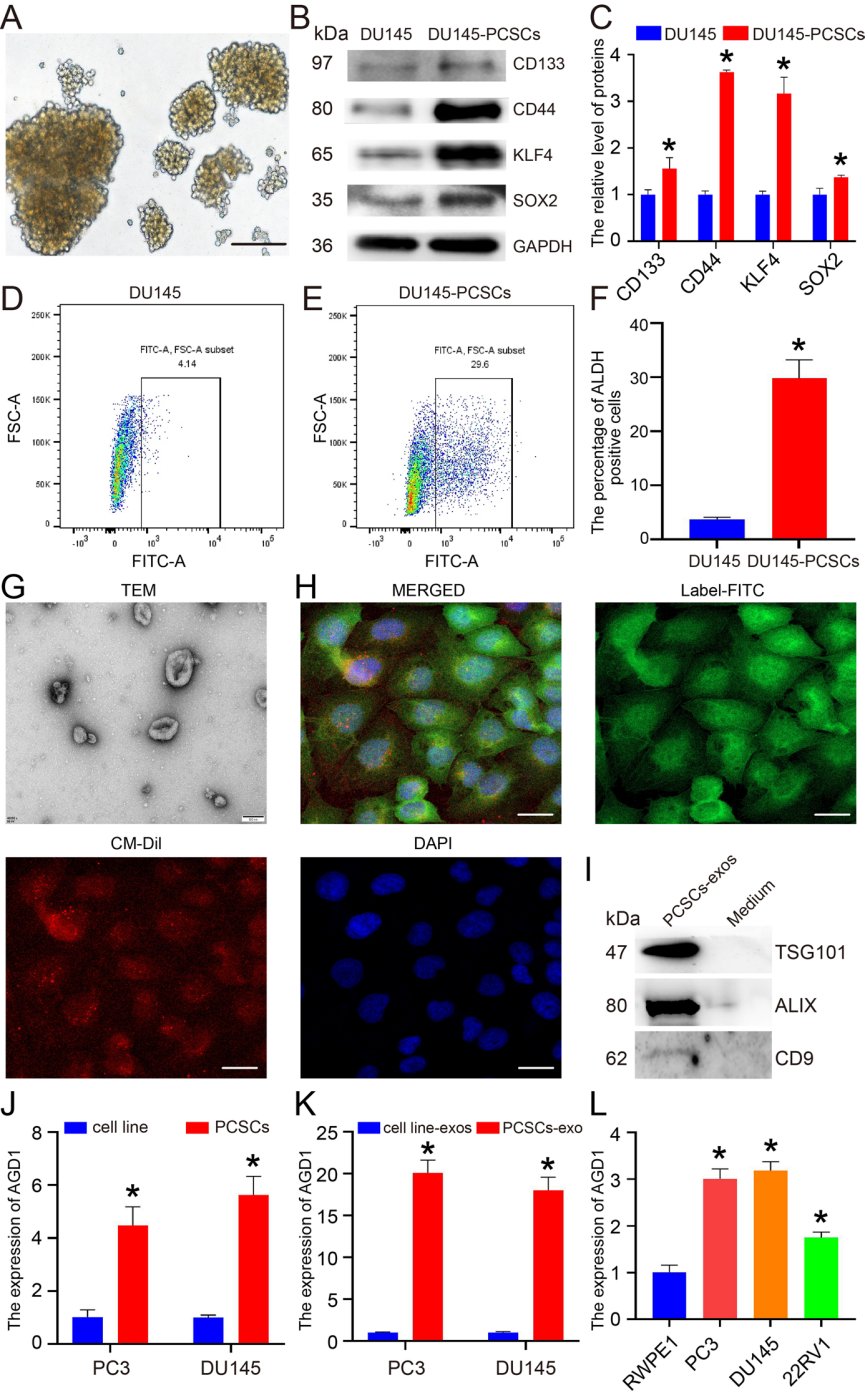
AGD1 was highly expressed in exosomes derived from PCSCs and PCSCs. **A**. DU145-PCSCs were obtained using serum-free suspension culture (scale bars = 100 μm). **B-C**. Western blots showing the expression of PCSCs markers CD133, CD44, KLF4, and SOX2. **D-F.** Flow cytometry analysis revealed the percentage of ALDH positive cells in DU145 cells and DU145-PCSCs. **G.** Exosomes were obtained from the culture medium derived from DU145-PCSCs (scale bars = 100 nm). **H.** CM-Dil tracer experiments showed the distribution of exosomes in DU145 cells (scale bars = 10 μm). **I.** Western blots showing the expression of TSG101, ALIX, and CD9 in DU145-PCSCs exosomes and DU145-PCSCs culture medium. **J-K.** Real-time PCR revealed the expression of AGD1 mRNA in PC3 vs. PC3-PCSCs and DU145 vs. DU145-PCSCs and their derived exosomes. **L.** Real-time PCR showed the levels of AGD1 mRNA in PC3, DU145, 22RV1, and RWPE-1 cells. Data are presented from three independent experiments. * indicates statistically significant differences (*p* < 0.05)

PC3 and DU145, two types of CRPC cells, were utilized in this research. PCSCs were obtained *via* serum-free media as previously described [29]. After two weeks of culture, DU145 cells formed clone spheres, designated as DU145-PCSCs (Fig. 1A). Western blot analysis revealed that DU145-PCSCs highly expressed stem cell markers, such as CD133, CD44, KLF4, and SOX2, compared to DU145 cells (Fig. 1B and C). Additionally, the expression of ALDH in DU145-PCSCs was higher than in DU145 cells according to the STEMCELL assay (Fig. 1D and F). Exosomes derived from DU145-PCSCs were isolated and identified. Transmission electron microscopy (TEM) analysis showed that the exosomes had a round-shaped appearance (Fig. 1G). CM-Dil tracer experiments indicated that labeled exosomes were distributed around the cytoplasm and nucleus of DU145 cells (Fig. 1H). Furthermore, western blot analysis demonstrated that the exosomes isolated from DU145-PCSCs expressed TSG101, ALIX, and CD9, which are specific markers of exosomes (Fig. 1I). Collectively, these results confirm the successful isolation of exosomes derived from DU145-PCSCs. Real-time PCR analysis showed that AGD1 was upregulated in PC3/DU145-PCSCs and their derived exosomes compared to PC3/DU145 cells (Fig. 1J and K). Additionally, AGD1 expression was higher in PCa cells compared to RWPE-1 cells, human normal prostate epithelial cell line (Fig. 1L). In summary, AGD1 was highly expressed in PC3/DU145-PCSCs and their exosomes, indicating that it may participate in CRPC progression.

### AGD1 derived from PCSCs decreased the sensitivity of docetaxel chemotherapy in CRPC

**Fig. 2:**
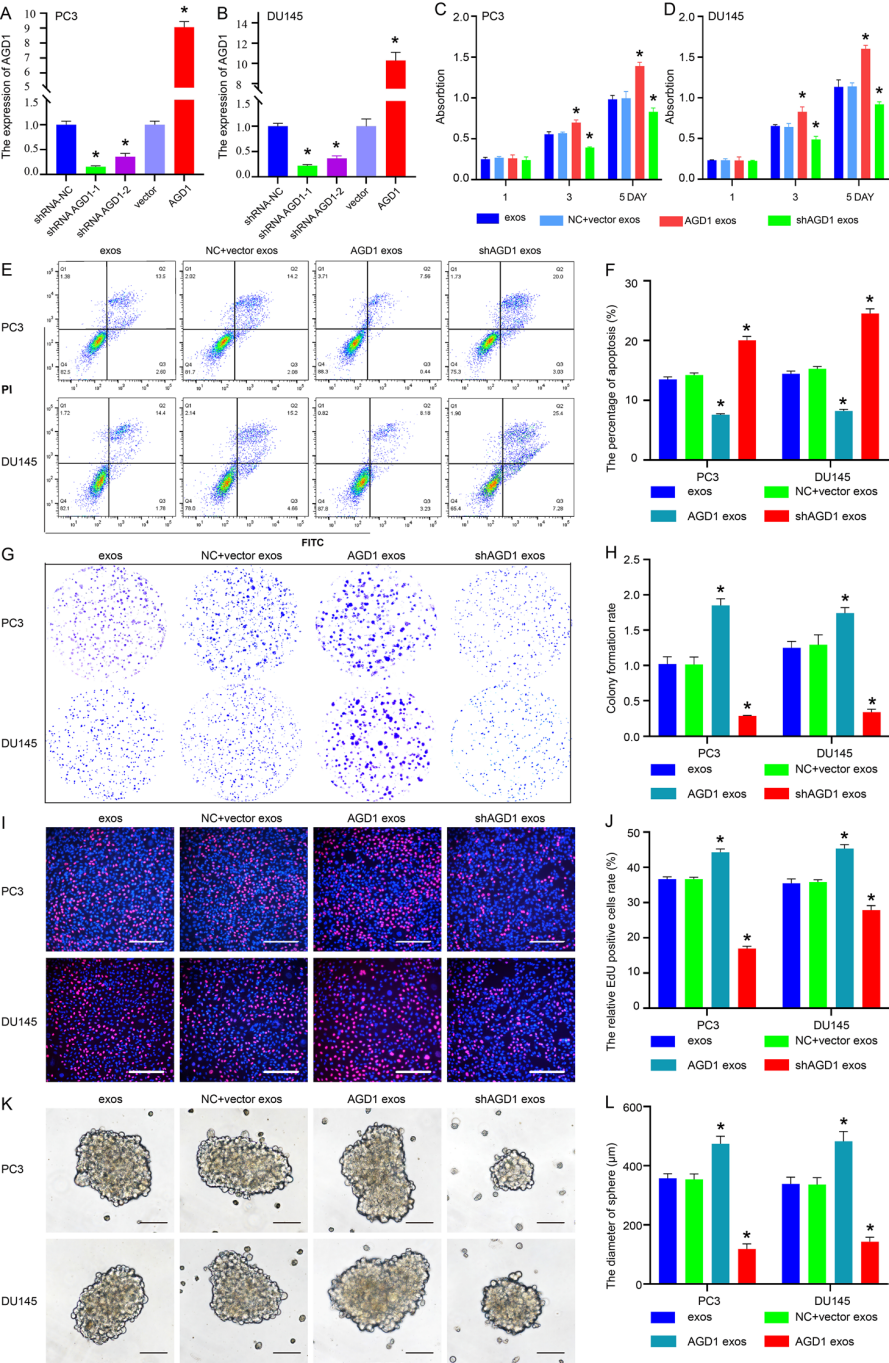
AGD1 derived from PCSCs exosomes decreased the sensitivity of docetaxel chemotherapy in CRPC. Exosomes were isolated from PCSCs, shRNA NC plus vector-PCSCs, AGD1-PCSCs, and shAGD1-PCSCs to assess the impact of exogenous AGD1 on docetaxel therapy in CRPC. **A-B.** Real-time PCR demonstrated the knockdown or overexpression efficiency of AGD1 in PC3 and DU145 cells using shRNA or overexpression plasmids. **C-D.** Cell proliferation assay revealed changes in the proliferation ability of PC3 and DU145 cells after treatment with exosomes. **E-F.** Annexin V-FITC/PI assay showed changes in the percentage of apoptotic PC3 and DU145 cells after treatment with exosomes. **G-H.** Colony formation ability of PC3 and DU145 cells after treatment with exosomes. **I-J.** EdU assay indicated alterations in EdU incorporation in PC3 and DU145 cells after treatment with exosomes (scale bars = 100 μm). **K**,** L.** Stemness of PC3 and DU145 cells after treatment with exosomes (scale bars = 100 μm). Data are presented from three independent experiments. * indicates statistically significant differences (*p* < 0.05)

Recombinant pLenO-GTP-AGD1-overexpression plasmids and shRNA plasmids were used to examine the effect of AGD1 on docetaxel chemotherapy in CRPC. Real-time PCR showed that AGD1 expression was significantly upregulated after transfection with AGD1 plasmids in PC3 (9.07 ± 0.37 vs. 1.00 ± 0.07) and DU145 cells (10.29 ± 0.80 vs. 1.00 ± 0.15). Meanwhile, shRNA1-2 plasmids significantly downregulated AGD1 mRNA levels in CRPC cells, with shRNA1 being more efficient than shRNA2 (Fig. 2A and B). Consequently, shRNA1 (0.15 ± 0.02 vs. 1.00 ± 0.07 in PC3 cells; 0.22 ± 0.02 vs. 1.00 ± 0.06 in DU145 cells) was selected to investigate the function of low AGD1 expression in CRPC cells. We detected the half maximal inhibitory concentrations (IC_50_) of docetaxel in PC3 (35.38 ± 0.98 nM) and DU145 (31.83 ± 0.96 nM) (Fig. S1). Cell proliferation assay demonstrated that the proliferation of PCa cells treated with 20 nM docetaxel increased after incubation with exosomes from AGD1-overexpressing PCSCs (AGD1-OE-PCSCs-exos) on days 3 and 5 compared to the control group (PCSCs-exos) and decreased after incubation with exosomes from shAGD1 PCSCs (shAGD1-PCSCs-exos) (Fig. 2C and D). Annexin V-FITC/PI assay showed a decreased percentage of apoptotic PCa cells incubated with AGD1-OE-PCSCs-exos (7.58 ± 0.16% vs. 13.50 ± 0.40% in PC3; 8.21 ± 0.27% vs. 14.43 ± 0.45% in DU145) and an increased percentage with shAGD1-PCSCs-exos under 20 nM docetaxel (20.03 ± 0.65% vs. 13.50 ± 0.40% in PC3; 24.50 ± 0.82% vs. 14.43 ± 0.45% in DU145) (Fig. 2E and F). The colony formation assay indicated that PCa cells treated with 5 nM docetaxel and supplemented with AGD1-OE-PCSCs-exos formed more colonies than those with PCSCs-exos (1.85 ± 0.10 vs. 1.02 ± 0.10 in PC3; 1.74 ± 0.08 vs. 1.25 ± 0.09 in DU145), while fewer colonies formed with shAGD1-PCSCs-exos (0.29 ± 0.01 vs. 1.02 ± 0.10 in PC3; 0.34 ± 0.04 vs. 1.25 ± 0.09 in DU145) (Fig. 2G and H). EdU incorporation assay results demonstrated that the percentage of EdU-positive cells incubated with AGD1-OE-PCSCs-exos was higher than with PCSCs-exos (44.27 ± 0.96% vs. 36.7 ± 0.50% in PC3; 45.30 ± 1.18% vs. 35.47 ± 1.25% in DU145) and lower with shAGD1-PCSCs-exos (16.93 ± 0.65% vs. 36.7 ± 0.50% in PC3; 27.87 ± 1.29% vs. 35.47 ± 1.25% in DU145) (Fig. 2I and J). Furthermore, the diameter of PCa cell spheres significantly increased after treatment with AGD1-OE-PCSCs-exos (474.00 ± 25.52 μm vs. 357.00 ± 15.52 μm in PC3; 482.00 ± 33.96 μm vs. 338.67 ± 22.72 μm in DU145) and decreased with shAGD1-PCSCs-exos (118.67 ± 25.52 μm vs. 357.00 ± 15.52 μm in PC3; 143.33 ± 33.96 μm vs. 338.67 ± 22.72 μm in DU145) (Fig. 2K and L). Collectively, these findings suggest that AGD1 derived from PCSCs enhances the stemness of PCa cells and inhibits the therapeutic effect of docetaxel.

### AGD1 decreased the sensitivity of docetaxel chemotherapy in CRPC

**Fig. 3:**
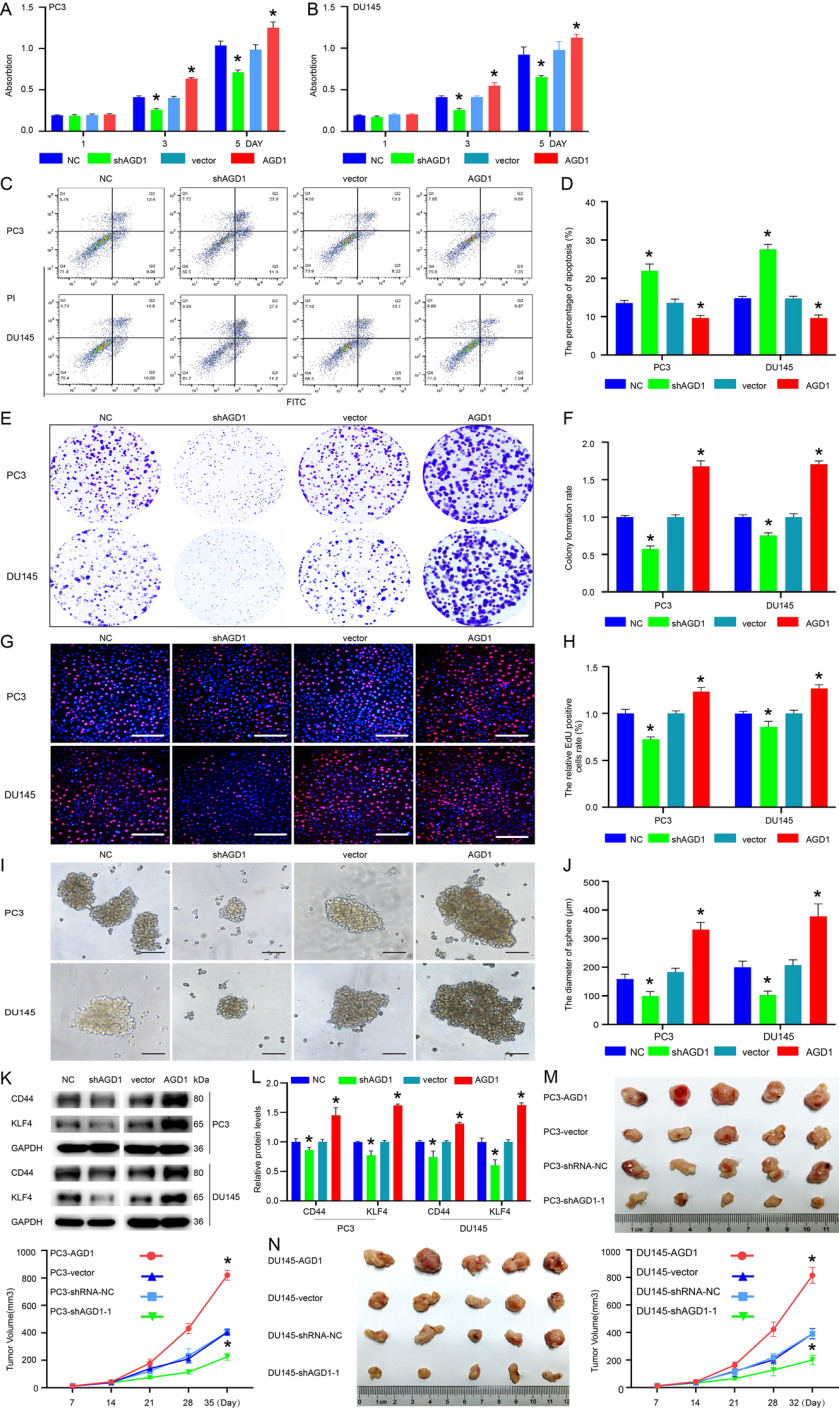
Endogenous AGD1 diminished the sensitivity of docetaxel chemotherapy in CRPC. AGD1 was silenced or overexpressed in CRPC cells to assess the influence of endogenous AGD1 on docetaxel efficacy. **A-B.** Cell proliferation assay revealed changes in the proliferation ability of PC3 and DU145 cells after AGD1 knockdown or overexpression. **C**,** D.** Annexin V-FITC/PI assay showed changes in apoptosis rates of PC3 and DU145 cells after AGD1 silencing or overexpression. **E**,** F.** Colony formation assay demonstrated the colony-forming ability of PC3 and DU145 cells with shAGD1 or AGD1 plasmids. **G**,** H**. EdU incorporation assay was performed to evaluate EdU incorporation in PC3 and DU145 cells following AGD1 knockdown or overexpression (scale bars = 100 μm). **I**,** J**,** K**,** L.** Stemness changes in PC3 and DU145 cells after AGD1 knockdown or overexpression (scale bars = 100 μm). **M-N.** In vivo assay displayed the therapeutic effect of docetaxel after AGD1 knockdown or overexpression. Data are presented from three independent experiments. * indicates statistically significant differences (*p* < 0.05)

The function of endogenous and exogenous AGD1 in the therapeutic effect of docetaxel on CRPC cells was examined using shAGD1 and AGD1 overexpression plasmids. The IC_50_ of docetaxel in PC3 and DU145 treated cells was showed in Fig. S1. The cell proliferation assay indicated that proliferation decreased following AGD1 knockdown in CRPC cells treated with docetaxel, whereas it increased with AGD1 overexpression (Fig. 3A and B). The Annexin V-FITC/PI assay revealed a significant increase in apoptosis after AGD1 knockdown in docetaxel-treated PCa cells (22.03 ± 1.72% vs. 13.53 ± 0.71% in PC3 cells; 27.57 ± 1.30% vs. 14.83 ± 0.45% in DU145 cells), while AGD1 overexpression significantly decreased apoptosis (9.70 ± 0.60% vs. 13.60 ± 0.99% in PC3 cells; 9.67 ± 0.78% vs. 14.73 ± 0.57% in DU145 cells) (Fig. 3C and D). Similarly, colony formation was diminished by AGD1 knockdown and enhanced by AGD1 overexpression under docetaxel treatment (Fig. 3E and F). The EdU incorporation assay showed that AGD1 knockdown reduced the percentage of EdU-positive cells (0.73 ± 0.02 vs. 1.00 ± 0.04 in PC3 cells; 0.86 ± 0.06 vs. 1.00 ± 0.02 in DU145 cells), while AGD1 overexpression increased it (1.23 ± 0.04 vs. 1.00 ± 0.03 in PC3 cells; 1.23 ± 0.04 vs. 1.00 ± 0.02 in DU145 cells) under docetaxel treatment (Fig. 3G and H). Further analysis of AGD1’s impact on PCa stemness showed that prostasphere diameters decreased from 159.00 ± 16.52 μm to 99.67 ± 15.54 μm in PC3 cells and from 103.00 ± 14.00 μm to 70.33 ± 13.11 μm in DU145 cells with AGD1 knockdown. Conversely, AGD1 overexpression increased prostasphere diameters from 184.00 ± 12.77 μm to 331.67 ± 25.58 μm in PC3 cells and from 208.00 ± 18.08 μm to 378.33 ± 43.65 μm in DU145 cells (Fig. 3I and J). Western blot analysis demonstrated that CD44 and KLF4 expression was downregulated following AGD1 knockdown and upregulated after AGD1 overexpression (Fig. 3K and L). In vivo assays showed that the volume of subcutaneous xenograft tumors decreased with shAGD1 treatment and increased with AGD1 overexpression when treated with docetaxel twice weekly (Fig. 3M and N). Collectively, both endogenous and exogenous AGD1 enhanced the stemness of PCa cells and contributed to docetaxel resistance in CRPC.

### AGD1/USP10/METTL13 complexes decreased the sensitivity of docetaxel chemotherapy in CRPC through a mechanistic pathway

**Fig. 4:**
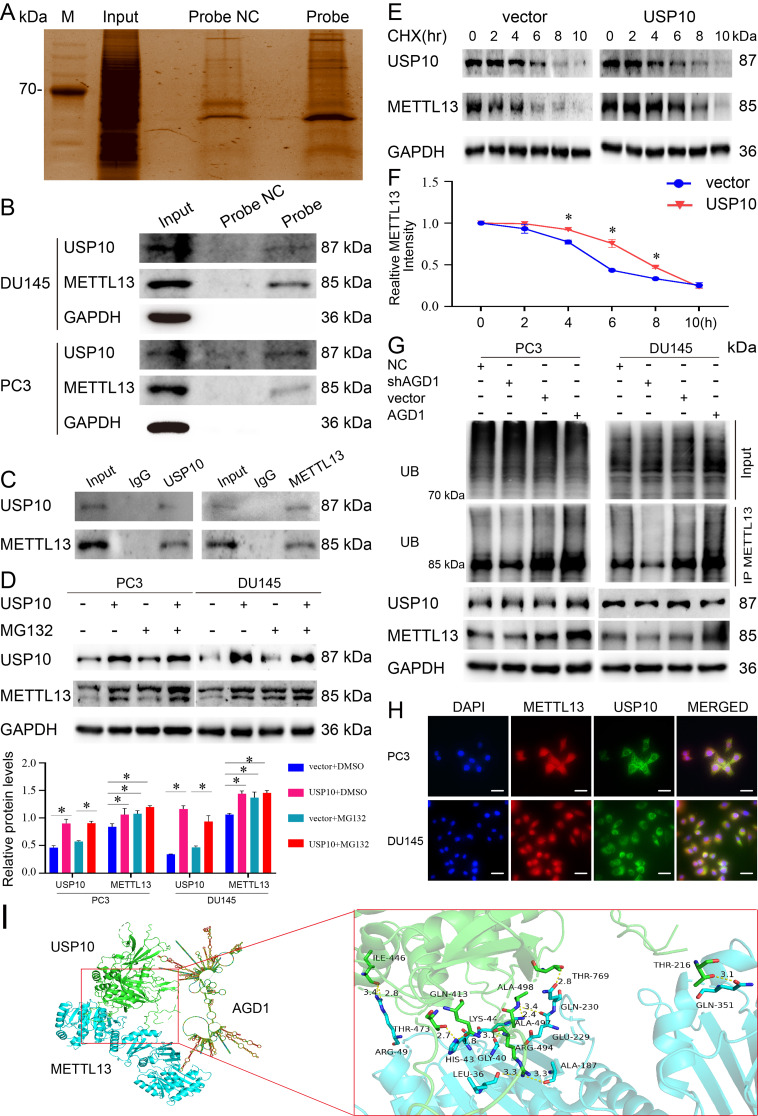
AGD1/USP10/METTL13 complexes diminished the sensitivity of docetaxel chemotherapy in CRPC through a mechanistic pathway. **A-B**. RNA pull-down assay combined with MS and western blot assays indicated that AGD1 associates with USP10 and METTL13 to form complexes. **C.** Co-IP assay demonstrated the interaction between USP10 and METTL13. **D.** Ubiquitination assay revealed that METTL13 expression was upregulated following USP10 overexpression in PC3 and DU145 cells, with the proteasome inhibitor MG132 producing a similar effect. **E-F.** Cycloheximide (CHX) assay showed that the half-life of METTL13 was significantly extended after USP10 overexpression in PC3 cells. **G.** co-IP assay indicated that AGD1 regulated the ubiquitination level of METTL13, and western blot analysis indicated that METTL13 expression decreased with AGD1 downregulation and increased with AGD1 overexpression, while USP10 expression remained unchanged with AGD1 alteration in PC3 and DU145 cells. **H.** Immunohistochemistry (IHC) revealed that USP10 is localized in the cytoplasm, whereas METTL13 is distributed in both the cytoplasm and nucleus (scale bars = 100 μm). **I.** Additionally, the docking score between USP10 and METTL13. Data are presented from three independent experiments. * indicates statistically significant differences (*p* < 0.05)

The RNA pull-down assay was employed to explore AGD1’s molecular mechanism in docetaxel treatment resistance (Fig. 4A). Western blotting and MS experiments (Supplementary Table. S3 for NC probe binding proteins, and Supplementary Table. S4 for AGD1 binding proteins) revealed that AGD1 could pull down USP10 and METTL13 proteins in PC3 and DU145 cells (Fig. 4B). Additionally, the co-IP assay demonstrated that USP10 formed complexes with METTL13 to regulate the therapeutic effect of docetaxel (Fig. 4C). USP10, a de-ubiquitinating enzyme, plays a vital role in removing ubiquitin from substrates, thereby promoting substrate protein stability. After transfecting PC3 and DU145 cells with USP10 plasmids, western blot analysis showed a significant upregulation of METTL13. Similarly, MG132 treatment also increased METTL13 expression (Fig. 4D). These results indicate that USP10 promotes METTL13 stabilization through de-ubiquitination. The cycloheximide assay further demonstrated that USP10 facilitates METTL13 stability (Fig. 4E and F). Additionally, the co-IP assay and western blots revealed that endogenous AGD1 regulates METTL13 ubiquitination levels (Fig. 4G), highlighting AGD1’s crucial role in the ubiquitination modification of METTL13. AGD1 positively correlates with METTL13 expression. Overall, AGD1/USP10/METTL13 complexes contribute to docetaxel treatment resistance in CRPC through METTL13 de-ubiquitination. Furthermore, double immunofluorescence showed that METTL13 and USP10 are involved in the biological behavior of CRPC cells in the nucleus (Fig. 4H). Finally, the docking score between USP10 and METTL13 was − 237.47 with an 85% confidence score (http://hdock.phys.hust.edu.cn/), indicating a stable interaction **(**Fig. 4I**)**.

### METTL13 decreased the docetaxel therapeutic effect in CRPC

**Fig. 5:**
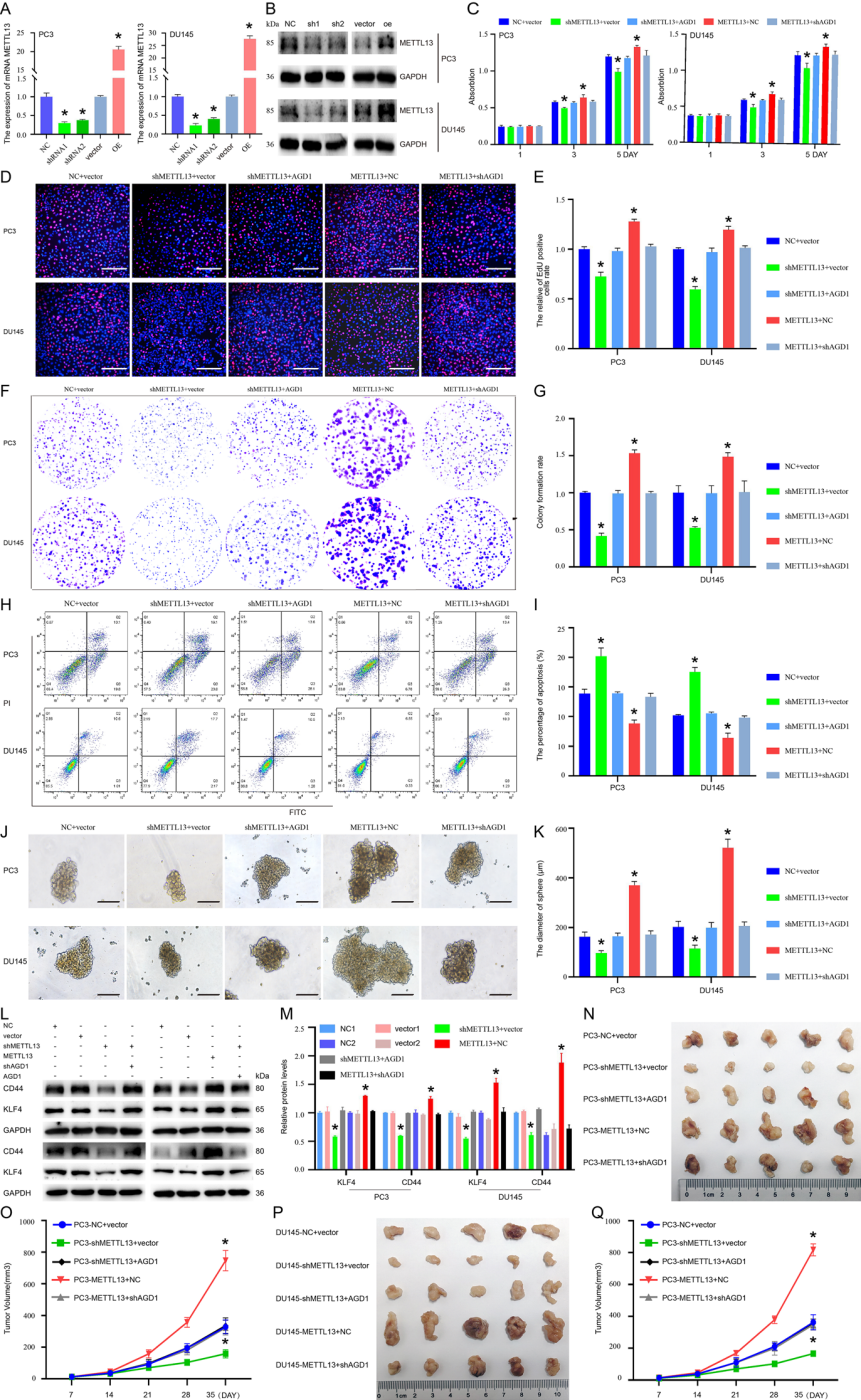
METTL13 reduced the therapeutic effect of docetaxel in CRPC cells. **A**. Real-time PCR showed the knockdown or overexpression efficiency of AGD1 in PC3 and DU145 cells using shRNA or overexpression plasmids. As shown in A, shRNA-1 significantly downregulated METTL13 expression, thus shRNA-1 was used to explore METTL13’s role in docetaxel efficacy in CRPC cells. B. Western blots confirmed successful silencing or overexpression of METTL13 in PC3 and DU145 cells. C. Cell proliferation assay demonstrated that knockdown of METTL13 could rescue the therapeutic effect of docetaxel diminished by AGD1 in CRPC, and vice versa. **D**,** E.** EdU assay indicated that METTL13 enhanced EdU incorporation in PC3 and DU145 cells, while AGD1 knockdown could mitigate this effect (scale bars = 100 μm). **F**,** G.** Plate clone formation assay suggested that clone formation ability increased with METTL13 overexpression, and this increase could be reversed by AGD1 knockdown in CRPC cells. **H**,** I.** Annexin V-FITC/PI assay demonstrated that METTL13 overexpression decreased apoptosis in PC3 and DU145 cells, and this decrease was reversed by shAGD1. **J-M.** Furthermore, METTL13 enhanced the stemness of PC3 and DU145 cells, and this enhancement could be reversed by shAGD1 (scale bars = 100 μm). **N-Q.** In vivo, the volume of subcutaneous tumors derived from PC3/DU145 cells increased with METTL13 overexpression, and this increase could be reversed by shAGD1. Data are presented from three independent experiments. * indicates statistically significant differences (*p* < 0.05)

To investigate the role of METTL13 in docetaxel resistance, stable PC3 and DU145 cell lines were established using METTL13 shRNA or overexpression plasmids. Real-time PCR and western blot analyses revealed that METTL13 shRNA-1 exhibited higher knockdown efficiency than shRNA-2 (Fig. 5A and B). Similarly, the IC_50_ value of docetaxel in PC3 and DU145 treated cells were showed in Fig. S1. Cell proliferation assay and EdU incorporation assays demonstrated that docetaxel-treated CRPC cells showed significantly increased proliferation following METTL13 overexpression, while proliferation decreased with METTL13 knockdown, and these effects were reversed by AGD1 (Fig. 5C, D and E). As illustrated in Fig. 5E, the percentage of EdU-positive cells rose with METTL13 overexpression (128 ± 2% vs. 100 ± 3% in PC3; 120 ± 4% vs. 100 ± 2% in DU145) and fell with METTL13 shRNA-1 (73 ± 4% vs. 100 ± 3% in PC3; 60 ± 3% vs. 100 ± 2% in DU145). Similarly, plate clonality assays indicated that colony formation rates significantly increased with AGD1 overexpression (153 ± 5% vs. 100 ± 2% in PC3; 149 ± 6% vs. 100 ± 10% in DU145) and decreased with AGD1 knockdown (42 ± 4% vs. 100 ± 2% in PC3; 53 ± 2% vs. 100 ± 10% in DU145) (Fig. 5F and G). Conversely, apoptotic cell levels decreased with METTL13 overexpression (8.86 ± 0.53% vs. 13.87 ± 0.75% in PC3; 6.43 ± 0.80% vs. 10.17 ± 0.15% in DU145) and increased with METTL13 shRNA-1 (20.17 ± 1.44% vs. 13.87 ± 0.75% in PC3; 17.53 ± 0.76% vs. 10.17 ± 0.15% in DU145) (Fig. 5H and I). Prostasphere formation assays showed that METTL13 overexpression enhanced the stemness of CRPC cells (371 ± 15 μm vs. 163 ± 18 μm in PC3; 522 ± 35 μm vs. 202 ± 23 μm in DU145), while METTL13 knockdown reduced it (97 ± 10 μm vs. 163 ± 18 μm in PC3; 115 ± 14 μm vs. 202 ± 23 μm in DU145) (Fig. 5J and K). Western blot analysis confirmed that KLF4 and CD44 were upregulated following METTL13 overexpression and downregulated with METTL13 knockdown in PC3 and DU145 cells (Fig. 5L and M). In vivo, subcutaneous tumor volume increased with METTL13 overexpression and decreased with shMETTL13 treatment and docetaxel (10 mg/kg) administered *via* intraperitoneal injection twice weekly (Fig. 5N, O, P and Q). Together, these findings suggest that METTL13 decreases the sensitivity of CRPC cells to docetaxel chemotherapy both in vitro and in vivo, with these effects being reversible by AGD1.

### CD44 was identified as the potential effector of METTL13

**Fig. 6:**
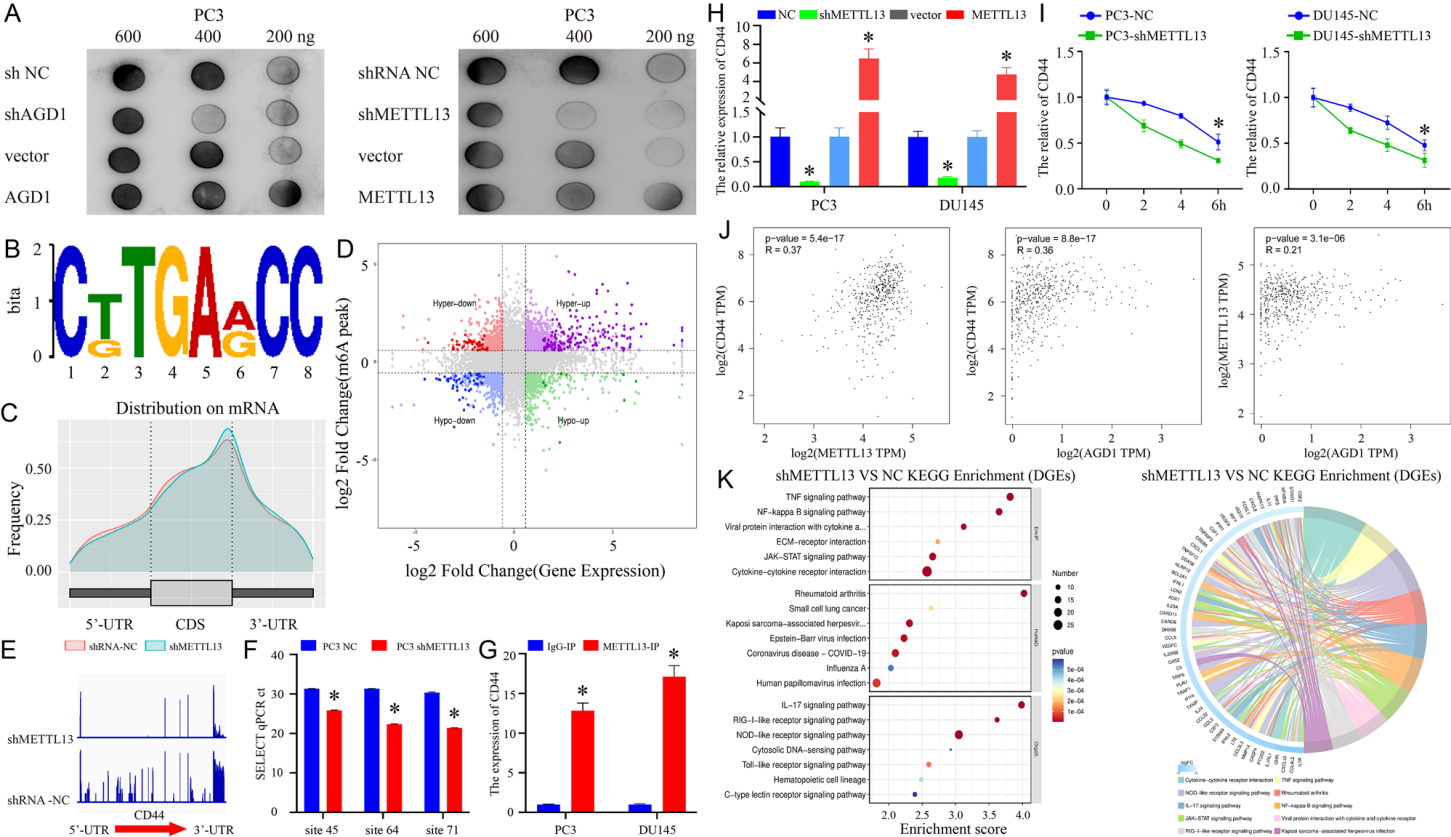
CD44 served as the effector of METTL13 in CRPC. **A**. Dot blot assay showed that total m6A levels were downregulated after AGD1 or METTL13 knockdown, while upregulated by AGD1 or METTL13 overexpression in PC3 cells. **B.** The m6A consensus sequence motif identified *via* MeRIP-seq in PC3 cells. **C.** Distribution of m6A peaks on mRNA in NC or shMETTL13 PC3 cells. **D.** MeRIP-seq combined with mRNA-seq revealed differential (hyper or hypo) m6A peaks (Y-axis) and differential (up or down) expression (X-axis) in shRNA METTL13 compared with NC group in PC3 cells (*p*-value < 0.05). **E.** MeRIP-seq identified the m6A modification site on CD44 mRNA in shRNA METTL13 and NC groups in PC3 cells. **F**. SELECT product at the 45, 64 and 71 site of CD44 3’ UTR with METTL13 interference and untreated cells. **G.** RIP assay indicated that CD44 was significantly enriched in the METTL13 antibody immunoprecipitation group compared to the IgG group. **H.** Real-time PCR showed that CD44 expression was positively correlated with METTL13 in PC3 and DU145 cells. **I.** RNA decay assay demonstrated that the half-life of CD44 mRNA was shortened by shMETTL13 in PC3 and DU145 cells. **J.** Bioinformatics analysis indicated a positive correlation between CD44, METTL13, and AGD1 expression (http://gepia.cancer-pku.cn/). **K.** KEGG analysis showed differential pathway enrichment after METTL13 knockdown in PC3 cells. Data are presented from three independent experiments. * indicates statistically significant differences (*p* < 0.05)

METTL13, an m6A “writer,” has been implicated in numerous diseases. This study explored changes in total m6A levels following AGD1 or METTL13 knockdown or overexpression. Dot blot assays revealed that m6A levels significantly decreased after AGD1 or METTL13 knockdown, while they increased with AGD1 or METTL13 overexpression in PC3 cells (Fig. 6A), and the RNA loading control was showed in Fig. S2. These results suggest that AGD1 regulates m6A modification *via* METTL13 in CRPC. Furthermore, MeRIP assays were utilized to identify potential effectors of METTL13 in CRPC. The m6A consensus motif, AGACA, was identified in PC3 cells (Fig. 6B), consistent with previous reports [38]. MeRIP assays indicated that m6A peaks were distributed at the 5’-UTR, coding sequence (CDS), and 3’-UTR of mRNAs (Fig. 6C). To investigate the underlying mechanisms of METTL13-dependent m6A modification, an integrated analysis combining MeRIP-seq and mRNA-seq was conducted using shMETTL13 and NC in PC3 cells. Figure 6D and Supplementary Table S5 show that among 252 genes, 156 had increased levels in both MeRIP-seq and mRNA-seq, 47 had decreased levels in both, 8 had decreased m6A levels and increased mRNA levels, and 41 had high m6A levels but low mRNA levels. Notably, m6A accumulation in the 3’-UTR regions of CD44 significantly decreased following METTL13 knockdown in PC3 cells (Fig. 6E). Furthermore, SELECT was executed to probe the m6A sites of CD44. SRAMP (http://www.cuilab.cn/sramp) was utilized to predict the potential m6A sites at the CD44 3’UTR. As those results, the N6-methyladenosine modification level at position 45, 64 and 71 was significantly decreased after downregulation METTL13, indicating those sites played a vital role in regulating CD44 methylated modification (Fig. 6F). Additionally, RIP assays demonstrated that anti-METTL13 interacted with CD44 mRNA at a high level compared to the IgG group in PC3 cells (Fig. 6G). Real-time PCR confirmed that CD44 expression markedly decreased with shMETTL13 and increased with METTL13 overexpression (Fig. 6H). RNA decay rate assays showed that the half-lives of CD44 mRNA were significantly shortened after METTL13 knockdown in PC3 and DU145 cells (Fig. 6I). Thus, METTL13 methylates CD44 mRNA, enhancing its stability, while METTL13 knockdown decreases CD44 mRNA stability. Bioinformatics analysis (GEPIA) demonstrated that AGD1 positively correlates with CD44 and METTL13 expression, and METTL13 similarly correlates with CD44 expression in patients with PCa (Fig. 6J). KEGG analysis of differentially expressed genes (DEGs) after METTL13 knockdown in PC3 cells revealed significant changes in the JAK-STAT and NFκB signaling pathways (Fig. 6K, Supplementary Table S6), which are crucial in determining the fate of prostate cancer stem cells [39–41].

### AGD1 activated the pSTAT3/PI3K-AKT pathway and diminished the therapeutic effect of docetaxel in CRPC in vivo

**Fig. 7:**
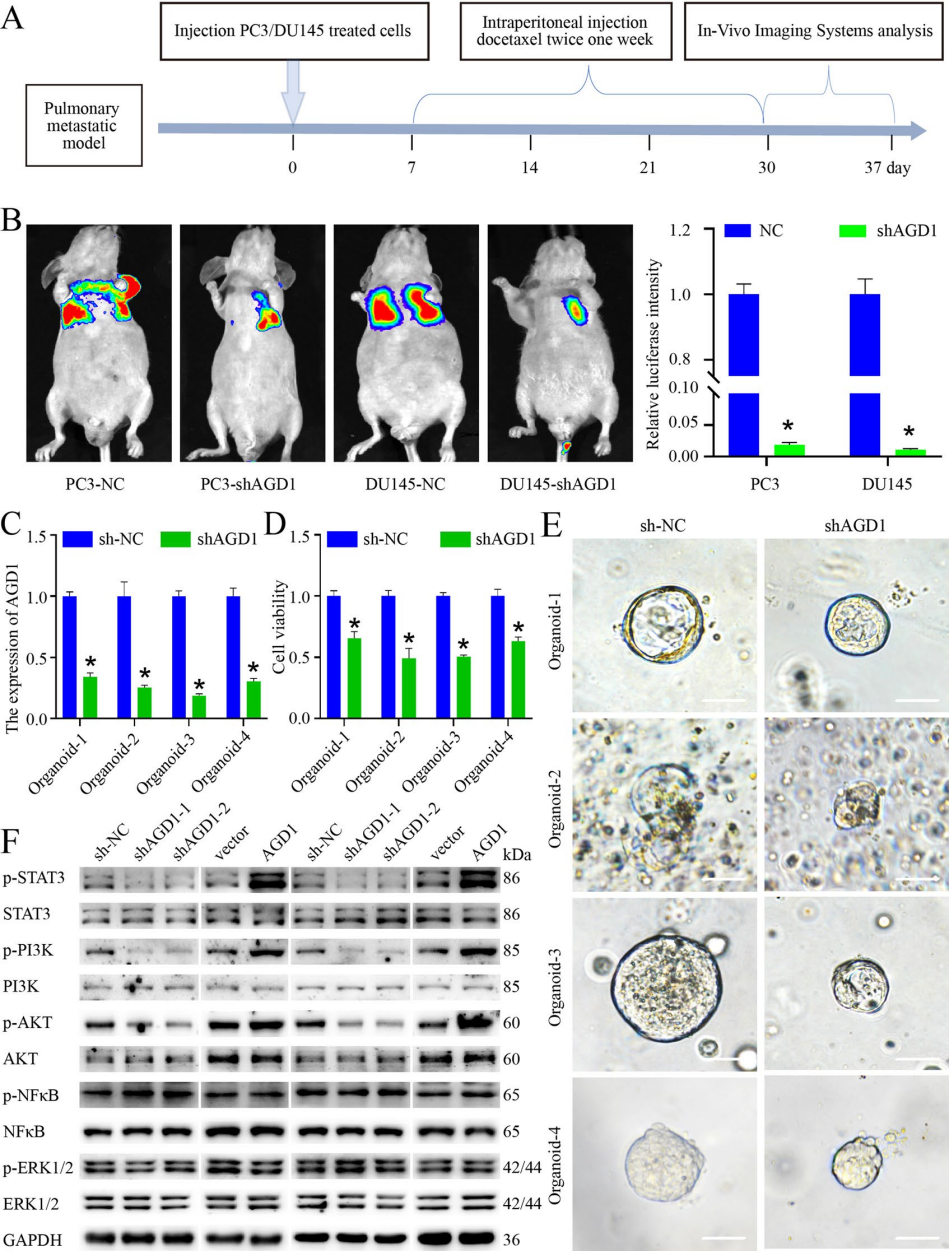
The AGD1/USP10/METTL13-pSTAT3/PI3K-AKT axis diminished the therapeutic effect of docetaxel in CRPC. **A**. Schematic illustration of the lung metastasis model. **B**. Lung metastasis model showed that the therapeutic effect of docetaxel was enhanced by shAGD1 in PC3 and DU145 cells. C. Real-time PCR confirmed successful silencing of AGD1 by shAGD1 in organoids derived from patients with CRPC. D. Cell viability was assessed after AGD1 knockdown in organoids on the 5th day. E. Morphological characteristics of organoids after AGD1 knockdown (scale bars = 100 μm). F. Western blots demonstrated that the pSTAT3/PI3K-AKT pathway was activated by AGD1, while the NFκB and ERK pathways remained unchanged. Data are presented from three independent experiments. * indicates statistically significant differences (*p* < 0.05)

A lung metastasis model was used to investigate the chemosensitivity to docetaxel following AGD1 knockdown. As shown in Fig. 7A and B, the therapeutic effect of docetaxel was significantly enhanced after AGD1 knockdown in PC3 and DU145 cells, resulting in a marked reduction in metastatic foci. Additionally, an organoid model was employed to further examine the therapeutic effect of docetaxel. Organoids 1, 2, 3, and 4 were derived from patients with CRPC resistant to docetaxel treatment. Real-time PCR confirmed that AGD1 expression was significantly silenced by shRNA (Fig. 7C). The cell proliferation assay demonstrated a reduction in cell viability in shAGD1-treated organoids with docetaxel on the fifth day (Fig. 7D). Moreover, the diameter of the organoids was significantly reduced following AGD1 knockdown (Fig. 7E). Additionally, changes in signaling pathways were investigated after AGD1 knockdown or overexpression in PC3 and DU145 cells. Western blot analysis revealed significant alterations in the pSTAT3/PI3K-AKT pathway following AGD1 knockdown or overexpression, while the NFκB and ERK1/2 pathways remained largely unchanged (Fig. 7F). Collectively, these results suggest that AGD1 knockdown enhances the therapeutic effect of docetaxel in CRPC *via* the pSTAT3/PI3K-AKT signaling pathway.

### Liposomal-chitosan/siAGD1 nanocomplexes enhanced the therapeutic effect of docetaxel in CRPC in vivo

**Fig. 8:**
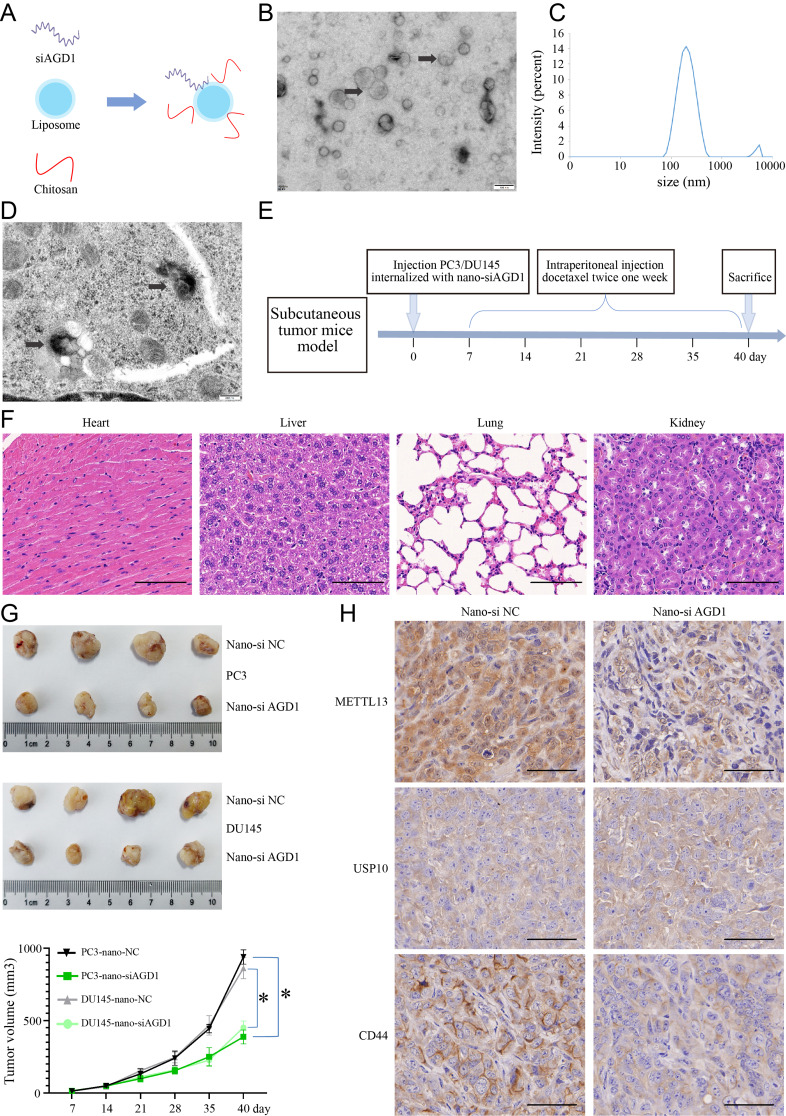
Liposomal-chitosan/siAGD1 nanocomplexes drug delivery systems enhanced the therapeutic effect of docetaxel in CRPC in vivo. **A**. Schematic illustration of the liposomal-chitosan/siAGD1 nanocomplexes. **B.** Transmission electron microscopy (TEM) image of liposomal-chitosan/siAGD1 nanocomplexes (scale bars = 100 nm). **C** Size distribution of liposomal-chitosan/siAGD1 nanocomplexes. **D.** Magnified TEM image of liposomal-chitos an/siAGD1 nanocomplexes in PC3 cells (scale bars = 200 nm). **E.** Graphical illustration of the nude mouse subcutaneous xenograft model with bi-weekly docetaxel injections. **F.** HE staining showed pathological changes in the heart, liver, lung, and kidney after injection of PC3 cells internalized with nano-siAGD1 and treated with docetaxel in vivo (scale bars = 100 μm). **G.** Volume of subcutaneous tumors derived from PC3/DU145 cells internalized with nano-siAGD1/nano-NC. **H.** METTL13 and CD44 expression correlated positively with AGD1, while USP10 expression was not associated with AGD1 (scale bars = 50 μm). Data are presented from three independent experiments. * indicates statistically significant differences (*p* < 0.05)

Liposomal-chitosan/siAGD1 nanocomplexes (nano-siAGD1) were synthesized as a promising therapeutic approach for CRPC to investigate the effect of docetaxel following AGD1 downregulation (Fig. 8A). Transmission Electron Microscopy (TEM) revealed that the nano-siAGD1 complexes were nearly spherical, with an average diameter of approximately 143 ± 3.6 nm (Fig. 8B and C). Successful internalization of nano-siAGD1 into PC3 cells was confirmed by TEM, consistent with a previous report [42] (Fig. 8D). The safety of nano-siAGD1 was assessed through subcutaneous injection in PC3/DU145 cells, with Hematoxylin-eosin (H&E) staining indicating no damage to major organs (Fig. 8E and F, Fig. S3). As shown in Fig. 8G, the volume of subcutaneous xenograft tumors derived from PC3/DU145-nano-siAGD1 cells was significantly reduced (from 938.67 ± 48.99 mm^3^ to 386.67 ± 48.29 mm^3^ in PC3, from 861.33 ± 72.67 mm^3^ to 451.67 ± 42.28 mm^3^ in DU145) with docetaxel treatment administered twice weekly. Immunohistochemistry (IHC) of the subcutaneous xenograft tumors from PC3-nano-siAGD1 demonstrated a dramatic downregulation of METTL13 and CD44 expression following AGD1 silencing, with no significant change in USP10 expression (Fig. 8H). These findings suggest that AGD1 represents a promising therapeutic target in CRPC.

## Discussion

This study demonstrated that AGD1 is highly expressed in exosomes derived from PCSCs. Functional experiments showed that both exogenous and endogenous AGD1 reduced the therapeutic efficacy of docetaxel in CRPC by promoting the stemness of CRPC cells in vitro and in vivo. Mechanistically, RNA pull-down, co-IP, and MS assays revealed that AGD1, USP10, and METTL13 form complexes to regulate docetaxel chemotherapeutic efficacy in CRPC. USP10 was identified as a deubiquitinase for METTL13, leading to METTL13 protein accumulation. Additionally, MeRIP-seq combined with mRNA-seq suggested that CD44 is an effector of METTL13. Specifically, SELECT assays demonstrated that METTL13 promotes m6A accumulation at the 45, 64 and 71 sites of CD44 3’ UTR, lengthening the half-life of CD44, a crucial stem cell marker. KEGG analysis and western blots indicated that AGD1 reduces docetaxel’s therapeutic effect in CRPC by activating the pSTAT3/PI3K-AKT signaling pathway. Furthermore, the PCa organoid model and liposomal-chitosan/siAGD1 nanocomplexes drug delivery systems demonstrated that AGD1 knockdown significantly enhances docetaxel’s chemotherapeutic efficacy in CRPC. In conclusion, these findings elucidate the molecular mechanisms by which AGD1 influences the therapeutic effect of docetaxel in CRPC and suggest AGD1 as a promising candidate for therapeutic intervention in CRPC.

PCSCs, characterized by self-renewal capability and multilineage differentiation potential, have garnered increasing attention [43]. However, PCSCs lack specific markers, making it challenging to isolate distinct sub-populations completely. To address this, serum-free DMEM/F12 medium supplemented with epidermal growth factor (EGF), basic fibroblast growth factor (bFGF), bovine serum albumin (BSA), insulin, and N2 nutrients was utilized to culture PCSCs [29]. In our study, PCSCs were successfully derived from DU145 and PC3 cells using this serum-free medium, as evidenced by the high expression of stem cell markers CD44, CD133, KLF4, SOX2, and ALDH1. Additionally, these cells demonstrated prostasphere formation ability, a unique characteristic of stem cells. Exosomes were subsequently isolated from PCSCs using ultracentrifugation. TEM and western blot analyses confirmed the presence of exosome-specific proteins such as TSG101, ALIX, and CD9, consistent with previous reports [44]. Increasing researches have shown that exosomes derived from CSCs transfer bioactive molecules to the tumor microenvironment *via* paracrine or distant secretion, aiding cancer cells in evading immune surveillance [45]. For instance, LncRNA H19 from gefitinib-resistant human non-small cell lung cancer cell exosomes can transfer gefitinib resistance to non-resistant NSCLC cells [46]. Similarly, exosomes from CD103+-CSCs promote metastasis in clear-cell renal cell carcinoma by delivering miR-19b-3p [47]. Another study demonstrated that agrin protein derived from extracellular vesicles of pancreatic cancer stem cells promotes YAP activation *via* LRP-4, aiding non-stem cancer cells in adaptation and proliferation, while anti-agrin therapies offer targeted treatment for patients with pancreatic ductal adenocarcinoma [48]. In this study, AGD1 was found to be highly expressed in PCSCs and their exosomes, suggesting its crucial role in PCSCs. Both PCSCs-derived exosomes and endogenous AGD1 were observed to enhance the stemness of PCa cells and reduce the therapeutic efficacy of docetaxel.

Ubiquitination and de-ubiquitination are post-translational modification processes that regulate protein stability through a highly conserved ubiquitin system [49]. Ubiquitination involves three types of enzymes: ubiquitin-activating enzyme (Uba, E1), ubiquitin-conjugating enzyme (UBC, E2), and ubiquitin ligase (E3), which transfer ubiquitin to target proteins in a cascade. De-ubiquitination reverses this process through DUBs [50]. DUBs are classified into six classes: UCHs, USPs, OTUs, MJDs, JAMMs, and MCPIPs, with USPs being the most diverse family [50]. Evidence suggests that USP7, USP9X, USP10, USP22, and USP44 play roles in maintaining stem cell pluripotency [51, 52]. This study demonstrates that AGD1, USP10, and METTL13 form a complex that regulates docetaxel treatment resistance. USP10 deubiquitinates METTL13, leading to the stabilization and accumulation of METTL13 protein. Furthermore, m6A is the most common RNA modification in mammals, primarily accumulating in 3′ untranslated regions (3′UTRs) near stop codons with the consensus motif sequence 5’-RRACH-3’ [53]. Enzymes involved in m6A modification are classified into three types: “writers,” “erasers,” and “readers,” which maintain dynamic equilibrium. Previous reports indicate that m6A methylation participates in various biological processes, including the fate of cancer cells and stem cancer cells [54]. For instance, in epithelial ovarian cancer, the ALKBH5/HOXA10 feedback loop erases m6A from JAK2 mRNA, activating the JAK2/STAT3 signaling pathway and leading to cisplatin resistance [55]. In bladder cancer, WATP associated with circ0008399 promotes TNFα-induced protein 3 (TNFAIP3) mRNA stability *via* an m6A-dependent mechanism, inducing cisplatin resistance [56]. METTL13 specifically methylates eEF1AK55, increasing eEF1A’s intrinsic GTPase activity, which enhances translational output and tumorigenesis in pancreatic and lung tumors [57]. Moreover, METTL13/METTL14/WTAP complexes and YTHDF2 facilitate PIK3CB mRNA and protein expression levels, significantly promoting the proliferation and migration of PTEN-deficient pancreatic ductal adenocarcinoma cells [58]. SELECT was reported as a powerful tool to explore m6A metabolism since it can be used in combination with genetics methods to detect m6A-related enzyme functions on modifying a specific m6A site [37]. This study elucidates that METTL13 promotes the stemness of CRPC cells by enriching m6A at the CD44 3’ UTR site utilizing MeRIP and SELECT assays, thereby reducing the therapeutic efficacy of docetaxel in CRPC.

Chemotherapy drugs are essential in cancer treatment, yet serious side effects are inevitable during drug delivery to tumors. To enhance antitumor efficacy and reduce side effects, NDDS have garnered significant attention due to their stability, targeted delivery, and controlled release properties, resulting in fewer side effects [59, 60]. Chitosan, a natural polymer composed of partially acetylated (1–4)-2-amino-2-deoxy-D-glucan, offers biocompatible and biodegradable advantages [61]. Despite attempts with various synthetic and natural polymers to modify liposome surfaces, chitosan remains the most extensively used carrier [62]. Over the past decades, chitosan has been widely utilized in drug delivery because of its biocompatibility, biodegradability, non-immunogenicity, low toxicity, and mucoadhesiveness [63]. This study synthesized liposomal-chitosan and siAGD1 nanocomplexes (nano-siAGD1) to investigate the therapeutic effect of docetaxel following AGD1 downregulation in CRPC. The safety of nano-siAGD1 drug delivery systems was evaluated, showing them to be safe and effective. Moreover, human prostate cancer organoids were utilized to explore the function of AGD1 in the therapeutic effect of docetaxel in CRPC. The cell viability was significantly decreased after downregulation AGD1 with docetaxel. In summary, AGD1 downregulation enhances the therapeutic effect of docetaxel in CRPC in vivo, which may highlight a potential therapeutic target and improve life expectancy for CRPC patients.

## Conclusions

In summary, this study explored the function of AGD1 in mediating docetaxel sensitivity in CRPC for the first time. AGD1 mediates the stemness and apoptosis of PCSCs and promotes docetaxel treatment resistance by enhancing tumor growth and metastasis in vitro and in vivo. Mechanistically, AGD1 interacts with METTL13 and USP10, mediating the ubiquitination and stability of METTL13 at the protein level. Consequently, METTL13 enhances the stability of CD44 mRNA through m6A transcriptional modification. Furthermore, liposomal-chitosan/siAGD1 nanocomplexes drug delivery systems demonstrated that AGD1 knockdown significantly enhanced docetaxel’s chemotherapeutic efficacy in CRPC. This study provides novel insights into the molecular mechanisms underlying docetaxel chemotherapy via the AGD1/USP10/METTL13-CD44-pSTAT3/PI3K-AKT cascade, offering new targets for gene therapy in CRPC.

## Electronic supplementary material

Below is the link to the electronic supplementary material.

Supplementary Material 1

Supplementary Material 2

Supplementary Material 3

Supplementary Material 4

Supplementary Material 5

Supplementary Material 6

Supplementary Material 7

Supplementary Material 8

Supplementary Material 9

## Data Availability

All data relevant to the study are showed in the article or supplementary information.

## Data availability

The datasets are available from the corresponding author on reasonable request.
