# Supplementary figures and images for: AGD1/USP10/METTL13 complexes enhance cancer stem cells proliferation and diminish the therapeutic effect of docetaxel via CD44 m6A modification in castration resistant prostate cancer
Source: J Exp Clin Cancer Res. 2025 Jan 14;44:12. doi: 10.1186/s13046-025-03272-3 (PMC11730809; doi:10.1186/s13046-025-03272-3)

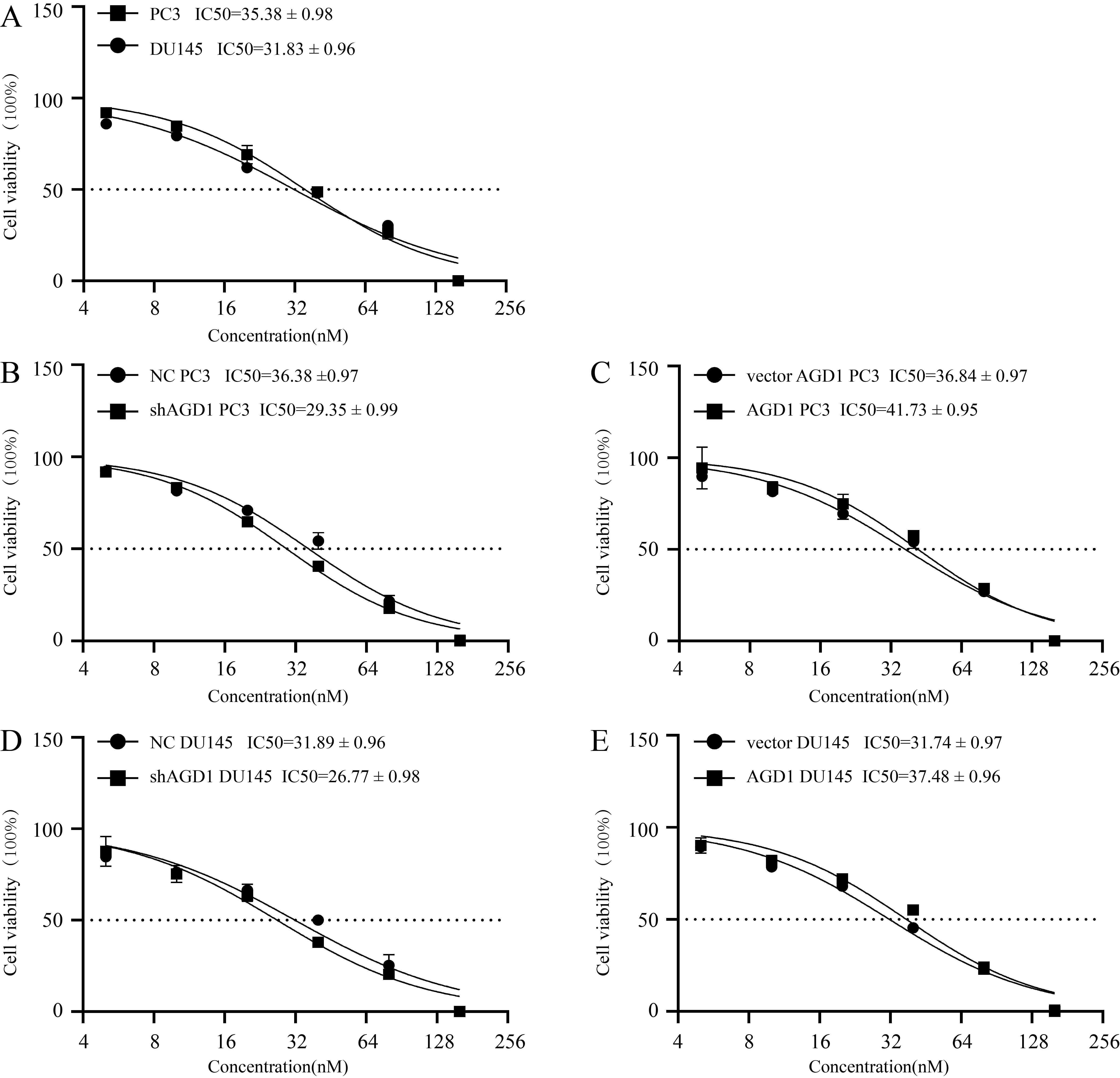

Supplement: Supplementary file 1 — Supplementary Material 1 [file 13046_2025_3272_MOESM1_ESM.jpg]

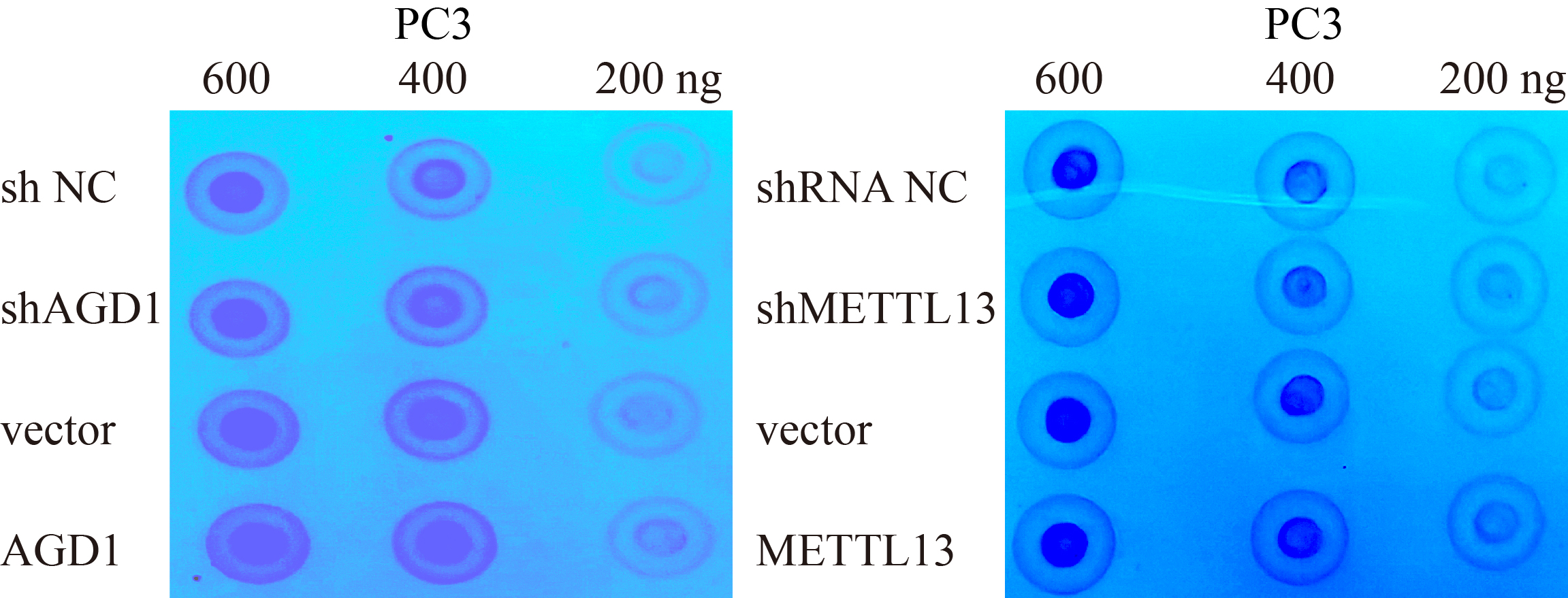

Supplement: Supplementary file 2 — Supplementary Material 2 [file 13046_2025_3272_MOESM2_ESM.jpg]

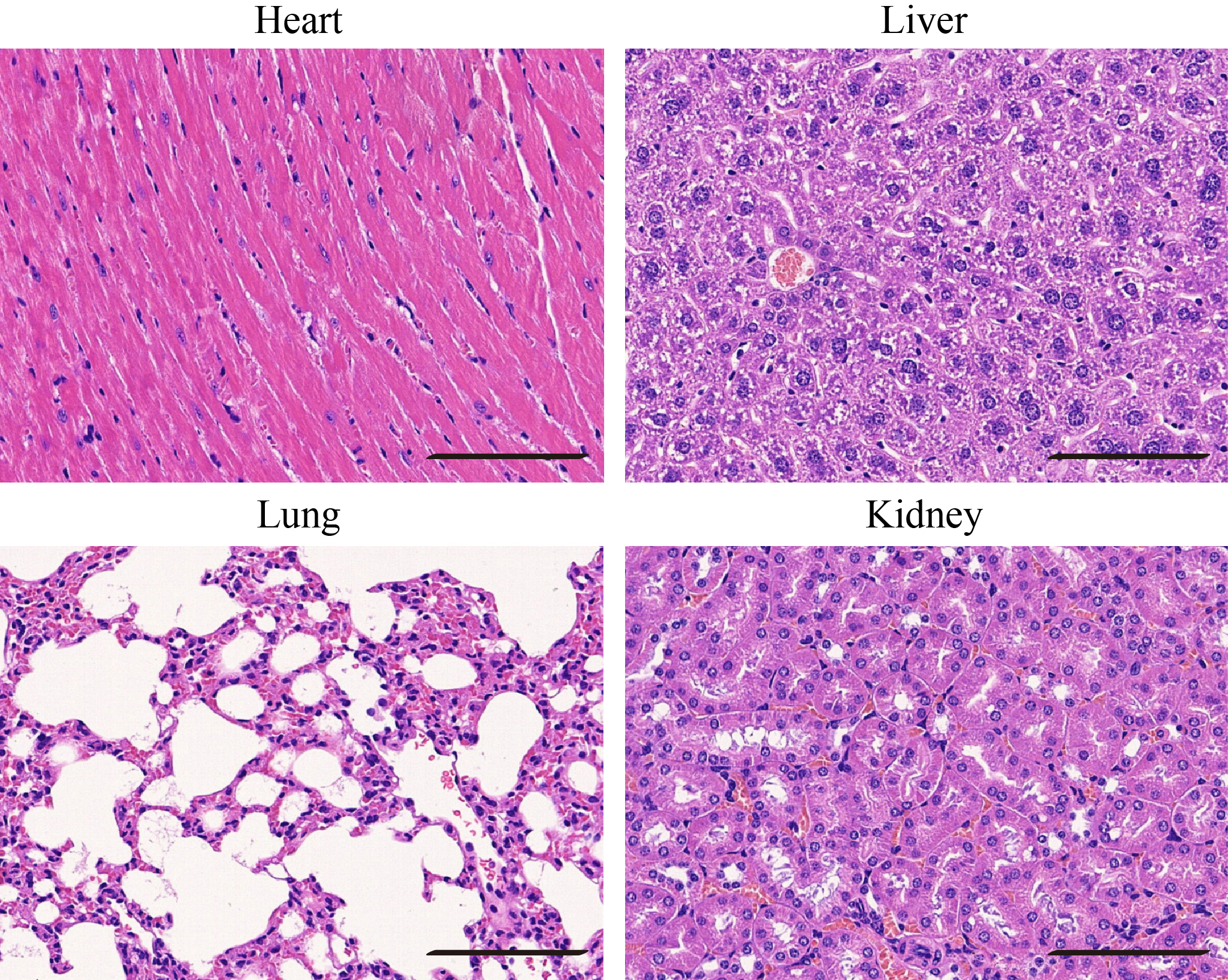

Supplement: Supplementary file 3 — Supplementary Material 3 [file 13046_2025_3272_MOESM3_ESM.jpg]
