# Supplementary material for: AGD1/USP10/METTL13 complexes enhance cancer stem cells proliferation and diminish the therapeutic effect of docetaxel via CD44 m6A modification in castration resistant prostate cancer
Source: J Exp Clin Cancer Res. 2025 Jan 14;44:12. doi: 10.1186/s13046-025-03272-3 (PMC11730809; doi:10.1186/s13046-025-03272-3)
Supplement: Supplementary file 5 — Supplementary Material 5 [file 13046_2025_3272_MOESM5_ESM.docx]

**Supplementary Table 2: siRNA/shRNA sequences, qRT-PCR primer sequences and pull-down probe sequences, primary antibodies**

**siRNA/shRNA sequences**

| **Genes** | **Sequences** |
| --- | --- |
| shRNA-NC | GATCTGTTCTCCGAACGTGTCACGTTTCAAGAGAACGTGACACGTTCGGAGAATTTTTTC |
| ShMIR100HG-1 | GCTAAGCTGGGTCTACTTATGCTCGAGCATAAGTAGACCCAGCTTAGCTTTTTT  TCCTCCCTGGTTTTTT |
| ShMIR100HG-2 | GGTTTGAGACACAATTCTTTCCTCGAGGAAAGAATTGTGTCTCAAACCTTTTTT |
| shMETTL13-1 | AATGTGGACCTTCATTCGGTC |
| shMETTL13-2 | TTGTGAGACACATCCTTCAGC |
| siRNA-NC | UUCUCCGAACGUGUCACGU |
| SiMIR100HG-1 | GCUAAGCUGGGUCUACUUAUG |

**qRT-PCR primer sequences**

| **Genes** | **Sequences (5'-3')** |
| --- | --- |
| MIR100HG-F | CCCAGTGCAAGGACAAAGA |
| MIR100HG-R | GCAGAGGAGGTGTCTTCAGG |
| METTL13-F | CGTGCGTTTGTCGTGTAAGG |
| METTL13-R | CATTTGAGGAGCTTGCCCAC |
| PROM1-F | AGTCGGAAACTGGCAGATAGC |
| PROM1-R | GGTAGTGTTGTACTGGGCCAAT |
| CD44-F | CTGCCGCTTTGCAGGTGTA |
| CD44-R | CATTGTGGGCAAGGTGCTATT |
| KLF4-F | CCCACATGAAGCGACTTCCC |
| KLF4-R | CAGGTCCAGGAGATCGTTGAA |
| GAPDH-F | GGAGCGAGATCCCTCCAAAAT |
| GAPDH-R | GGCTGTTGTCATACTTCTCATGG |

**Pull-down probe sequences**

| **Genes** | **Sequences** |
| --- | --- |
| NC | AAGCUACUGAUAGUGAACUGGAGGGC |
| MIR100HG | UUCGCAAAUAAGCGGAGCUUGGGAAG |

**Primary antibodies**

| **Antibody** | **Method** | **Dilution** | **Source** | **Catalog#** |
| --- | --- | --- | --- | --- |
| CD133 | WB | 1:1000 | Proteintech | 18470-1-AP |
| CD44 | WB | 1:1000 | CST | 96848 |
| SOX2 | WB | 1:1000 | CST | 3579 |
| KLF4 | WB | 1:1000 | CST | 4038 |
| TSG101 | WB | 1:1000 | Abcam | 125011 |
| ALIX | WB | 1:1000 | Abcam | 275377 |
| CD9 | WB | 1:1000 | Abcam | 236630 |
| Calnexin | WB | 1:1000 | Abcam | 22595 |
| METTL13 | WB | 1:1000 | Abcam | 186002 |
| METTL13 | ICC/IP | 1:100 | Abcam | 186002 |
| m6A | Dot blot | 1:5000 | Abcam | 208577 |
| USP10 | WB | 1:2000 | Proteintech | 67917-1-Ig |
| USP10 | ICC/IP | 1:100 | Proteintech | 67917-1-Ig |
| STAT3 | WB | 1:1000 | CST | 12640 |
| p-STAT3 | WB | 1:1000 | CST | 9145 |
| PI3K | WB | 1:1000 | CST | 9655 |
| p-PI3K | WB | 1:1000 | CST | 9655 |
| AKT | WB | 1:1000 | CST | 4691 |
| p-AKT | WB | 1:1000 | CST | 4060 |
| ERK1/2 | WB | 1:1000 | CST | 4695 |
| p-ERK1/2 | WB | 1:1000 | CST | 8544 |
| NFκB | WB | 1:1000 | CST | 6956 |
| p-NFκB | WB | 1:1000 | CST | 3039 |
| GAPDH | WB | 1:5000 | Abcam | 8245 |

**The potential m6A modification sites at the CD44 3' UTR**

| **Position** | **Sequence context** |
| --- | --- |
| 45 | UGGAA AGAAA CAACC GUUGG AA**A**CA UAACC AUUAC AGGGA GCUGG |
| 64 | AUAAC CAUUA CAGGG AGCUG GG**A**CA CUUAA CAGAU GCAAU GUGCU |
| 71 | UUACA GGGAG CUGGG ACACU UA**A**CA GAUGC AAUGU GCUAC |

**qRT-PCR primer sequences of SELECT**

| **Primer name** | **Primer sequences (5’ to 3’)** |
| --- | --- |
| CD44-site45 | F: TGGAAAGAAACAACCGTTGGA  R: TCGCAATGAAACAATCAGTAGCA |
| CD44-site64 | F: TGGAAAGAAACAACCGTTGGAA  R: CGCAATGAAACAATCAGTAGCAC |
| CD44-site71 | F: CTTGGAAAGAAACAACCGTTGGA |
|  | R: CGCAATGAAACAATCAGTAGCA |
